# Immune-Checkpoint Inhibitors for Biliary Tract Cancer: Who Benefits and What Is Next?

**DOI:** 10.3390/cancers17172811

**Published:** 2025-08-28

**Authors:** Lucía Ceniceros, Manuel de La Torre, Ana Landa Magdalena, Paloma Sangro, Josepmaria Argemí, Delia D’Avola, Bruno Sangro, Mariano Ponz-Sarvisé

**Affiliations:** 1 Liver Unit, Cancer Center Clinica Universidad de Navarra (CCUN), Program in Solid Tumors (CIMA), Universidad de Navarra, Clínica Universidad de Navarra, 31008 Pamplona, Spain; alandam@unav.es (A.L.M.); mponz@unav.es (M.P.-S.); 2 Liver Unit, Cancer Center Clinica Universidad de Navara (CCUN), Centro Investigación Biomédica en Red: Enfermedades Hepáticas y Digestivas (CIBEREHD), 31008 Pamplona, Spain; mdalaez@unav.es (M.d.L.T.); psangro@unav.es (P.S.); jargemi@unav.es (J.A.); ddavola@unav.es (D.D.); bsangro@unav.es (B.S.)

**Keywords:** immune-checkpoint inhibitors, biliary tract cancer, treatment outcome

## Abstract

Biliary tract cancer (BTC) is a rare and aggressive type of malignancy characterized by heterogeneity both in tumor biology and in the immune microenvironment. Most patients are diagnosed with advanced-stage disease and have limited curative options. Although the introduction of immune-checkpoint inhibitors (ICI) has transformed the treatment landscape for several solid tumors, their effects on BTC remain modest. New combinations have promoted incremental improvements in the survival of patients with advanced BTC, but the complex interplay between immune therapies and the tumor microenvironment continues to be a major challenge to improve therapeutic outcomes. Nonetheless, ongoing studies are investigating combinations that may potentially improve results in this lethal disease. This review provides an overview of the evolving role of ICIs in BTC, discusses the impact of tumor heterogeneity on treatment response, and explores future directions to optimize patient selection.

## Simple Summary

Biliary tract cancer remains one of the most challenging and deadly cancers, due to its silent progression and late diagnosis. It includes three different entities: intrahepatic cholangiocarcinoma, extrahepatic cholangiocarcinoma, which can be further subdivided into perihilar and distal subtypes, and gallbladder carcinoma. Only around 20% are eligible for surgery. Despite progress in recent years, the prognosis of patients with biliary tract cancer remains poor, and efforts to improve outcomes through novel combinations and treatment strategies are ongoing. The introduction of immune-checkpoint inhibitors combined with chemotherapy has recently established a new gold standard of care in the metastatic setting, and new combinations are being investigated in the neoadjuvant and adjuvant scenarios. Significant questions persist regarding how best to harness these therapies and which patients stand to benefit most. This review aims to provide a comprehensive overview of the role of immune-checkpoint inhibitors in biliary tract cancer. We highlight the impact of tumor heterogeneity, summarize clinical evidence supporting immune-checkpoint inhibitor-based regimens, and explore future directions to allow a better patient selection in future clinical trials aiming at improving treatment efficacy.

## 1. Introduction

BTC refers to a heterogeneous group of tumors, including intrahepatic cholangiocarcinoma (iCCA); extrahepatic cholangiocarcinoma (eCCA), which can be further subdivided into perihilar and distal subtypes; and gallbladder cancer (GB). These tumors are relatively rare. The incidence rate is below 6/100,000 individuals, accounting for approximately 3% of all gastrointestinal malignancies. Cholangiocarcinoma (CCA) mortality continues rising globally. Asian populations have the highest incidence rates compared in the West. In Western countries, deaths from iCCA are increasing and occur at a higher rate than deaths from eCCA. The epidemiology of CCA and GB varies significantly depending on the subtype, likely due to the influence of various risk factors. Chronic liver disease, such as cirrhosis or viral hepatitis, has been strongly associated with an increased incidence of iCCA [1]. This may partly explain why, in Western countries, iCCA-related mortality is rising more rapidly than eCCA-related mortality [2]. Another risk factor, such as diabetes, may also contribute to the global rise in these malignancies. Hypertension and obesity are considered two main risk factors of multiple malignancies; however, no significant associations were found either in iCCA or eCCA [2]. Most cancers of the BTC are adenocarcinoma arising from cholangiocytes. The main differential diagnosis for iCCA is metastases from adenocarcinoma of another origin and hepatocellular carcinoma. Immunohistochemical profile plays a pivotal role in the differential diagnosis of CCA, which typically shows positivity for cytokeratin 7 and cytokeratin 19, and cytokeratin 20 has a variable expression [3].

At presentation, evaluation of liver function in a blood analysis is fundamental. Imaging techniques are critical for positive and differential diagnosis, evaluating disease extension, and guiding therapeutic strategies. Thoracoabdominopelvic computed tomography remains the gold standard for staging lymph node and distant metastasis. For eCCA, including perihilar and distal subtypes, magnetic resonance imaging offers superior delineation of local extension in eCCA. Although [^18^F]-2-fluoro-2-deoxy-d-glucose-positron emission tomography (FDG-PET) is not routinely recommended for primary diagnosis, it can be valuable in detecting nodal metastases, distant metastases, and disease recurrence [4]. Surgery, including liver resection and transplantation, remains the only curative treatment option. However, recurrence rates are high, even after adjuvant therapy. On the other hand, approximately 80% of patients present with advanced-stage disease at diagnosis, with systemic palliative therapy as a unique treatment option. Recently, the introduction of chemotherapy and immunotherapy combinations has achieved a significant improvement in survival outcomes. Nevertheless, the prognosis of patients with BTC remains poor, with a median overall survival (OS) of approximately 1 year among patients with stage IV disease [4]. This review provides an overview of emerging perspectives on the treatment of BTC with immune-checkpoint inhibitors (ICIs).

## 2. Impact of Tumoral Immune Heterogeneity on Therapeutic Management of BTC

BTCs are characterized by a dense and desmoplastic stroma that can be attributed to an abundance of cancer-associated fibroblasts. Infiltration of innate and adaptive immune cells is also observed. Those cells can play tumor-promoting or tumor-suppressing roles depending on the context. Additionally, BTCs have a complex immune microenvironment with intricate and extensive crosstalk between various stromal and immune components [5]. This complex interplay significantly influences tumor progression and therapeutic response.

Tumor immune microenvironment heterogeneity is characterized by significant variations in the number, composition, and functional states of infiltrating immune cells across different tumors [6]. To better understand this complexity, extensive molecular studies using omics-based bulk tumor analyses have identified specific genetic alterations and oncogenic pathways, providing critical insights into the molecular basis of tumor heterogeneity [7].

Intratumoral heterogeneity may pose a significant barrier to therapeutic response. Tumor infiltration of adaptive immune system cells, particularly B and T lymphocytes, has been associated with a better prognosis among patients with BTC, whereas enrichment of innate immune cells, such as macrophages and neutrophils, has been correlated with poorer outcomes [8]. In a comprehensive molecular analysis of the tumor microenvironment in 566 patients with iCCA, distinct immune classes were revealed with potential therapeutic implications [7]. In particular, two notable subtypes were identified, namely the l2 and l4 immunogenic subtypes. The l2 immunogenic subtype was characterized by an inflammatory phenotype with diverse immune cell infiltration, suggesting a potentially effective antitumor immune response. The l4 mesenchymal subtype exhibited high levels of vascular factors, chemokines, and other paracrine mediators produced by activated fibroblasts, which enhanced protumorigenic pathways and restrained immune cell recruitment into the tumor tissue. This abundant fibrous stroma acted as a physical barrier, preventing immune cells’ access to tumor cells.

Intratumoral heterogeneity has been linked to cancer aggressiveness, which contributes to treatment resistance and impedes satisfactory therapeutic responses. Although differentiation into CD4+ and CD8+ T-cell lineages is considered irreversible, the cellular states within a cell type exhibit high plasticity, which complicates the understanding of the immune cell landscape in cancer [9]. A comprehensive study using single-cell transcriptomics to analyze biopsies from 37 liver cancer patients (25 hepatocellular carcinomas and 12 iCCA) before and after ICI therapy demonstrated a significant correlation between the number of clusters and patient outcomes. Tumors exhibiting a higher number of distinct cellular clusters were associated with shorter survival compared with those with a lower cluster number [10].

Knowledge of inter- and intratumoral heterogeneity in BTC could potentially impact treatment efficacy, particularly in the context of immunotherapy. These observations highlight the need for sophisticated approaches to characterize and eventually target diverse cellular populations within these complex malignancies. Insights into tumor subtypes, immune cell infiltration, and stromal characteristics could guide the selection of patients who may benefit from ICI or combination therapies. The search for predictive biomarkers for ICI efficacy and survival remains an advancing field. Despite the predictive value in other malignancies, programmed cell death protein ligand-1 (PDL-1) expression, microsatellite instability (MSI), or tumor mutational burden (TMB) often fail to capture the complex interactions within the tumor microenvironment (TME) in BTC. The generation of an immune-suppressive TME, frequently associated with upregulation of immune-checkpoint molecules on tumor cells, is one of the immune evasion strategies observed in MSI-high tumors. The anti-PD-1 pembrolizumab has been approved by the FDA for the treatment of patients with TMB-high (TMB ≥ 10 Mutations/Megabase) independent of tumor origin, based on biomarker analysis from the KEYNOTE-158 trial. Notably, this study did not include individuals with BTC. Trying to clarify if TMB is a good predictive biomarker in BTC population, a Phase II study evaluates the clinical activity of cisplatin–gemcitabine plus nivolumab; no correlation between TMB and progression-free survival (PFS) was observed [11]. While PDL-1 is an effective biomarker in multiple tumors, in BTC remains uncertain. Keynote-028 [12] and Keynote-158 [13] showed no significant differences in response rates according to PDL-1 status. Recent associations between specific genetic alterations, RNA expression patterns, treatment response, and prognosis were revealed in a study that integrated genomic and transcriptomic analysis. A multi-omics analysis reported that mutations in tumor protein p53 (TP53), breast cancer gene (BRCA2), and the cytokine pathway correlated with benefit from chemo-immunotherapy (chemo-IO), whereas Kirsten rat sarcoma (KRAS) G12D and at-rich interaction domain 1A (ARID1) loss-of-function mutations were associated with poorer clinical outcomes. Interestingly, high expression of cytotoxic T-lymphocyte antigen-4 (CTLA4) and CXCL9 predicted favorable responses to chemo-IO and improved survival [14]. Liquid biopsy techniques, particularly circulating tumor DNA (ctDNA), are emerging as promising non-invasive approaches for biomarker assessment. These methods may offer distinct advantages in BTC management by enabling real-time molecular surveillance and supporting adaptive therapeutic strategies. Discovering prognostic and treatment-predictive biomarkers is an active field of research.

## 3. Immune-Checkpoint Inhibitors

**Figure 1:**
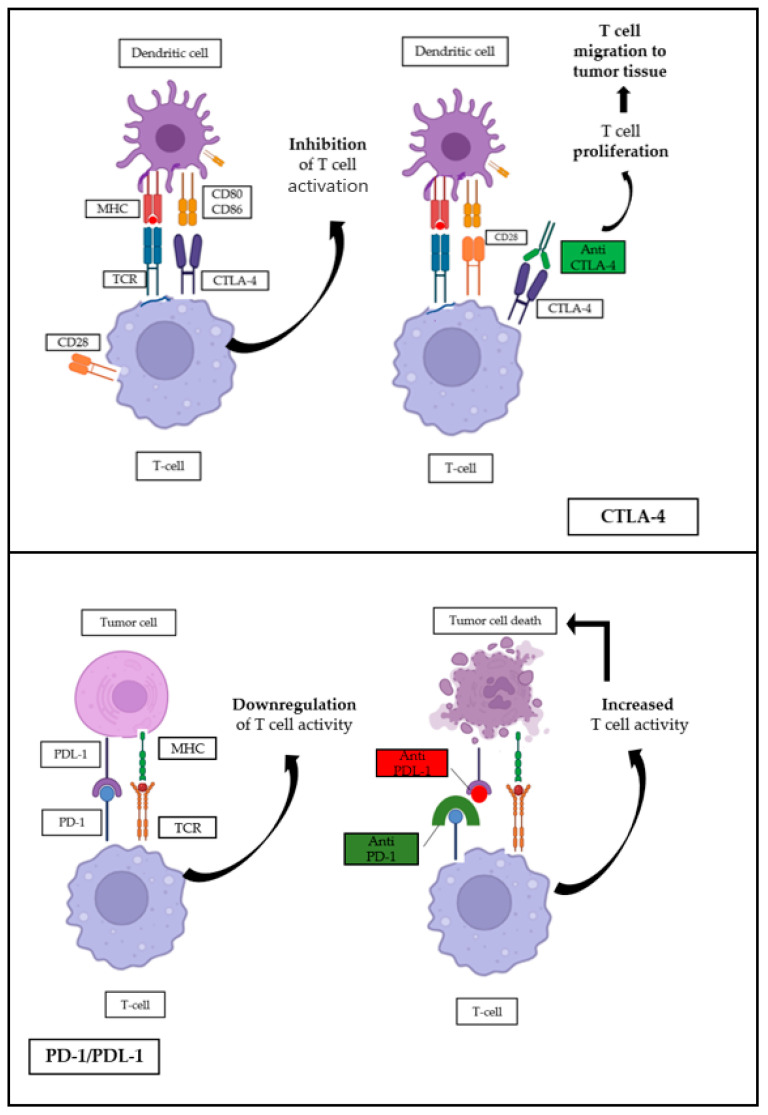
Anti-PD-1-PD-1/PDL-1 and Anti-CTLA-4 Mechanism of action.

The discovery of inhibitors to target checkpoint proteins, such as anti-programmed cell death protein-1/ligand-1 (PD-1/PDL-1) and CTLA-4, has revolutionized cancer immunotherapy and played a major role in the management of various solid tumors. These inhibitors can help elicit or reinforce the immune response against tumor cells [15]. The ICI mechanism of action involves a network of inhibitory and stimulatory pathways that govern immune cell activity. Engagement of PD-1 leads to reduced secretion of key cytokines, such as interleukin-2, interferon-γ, and tumor necrosis factor-α, and inhibits cell proliferation in part by interfering with the CD28 co-stimulatory signaling pathway. PD-1 interacts with two ligands: PDL-1 and PDL-2. PDL-1 is expressed on both tumor and immune cells. In certain malignancies, it serves as a predictive biomarker for response to anti-PD-1/PDL-1 antibodies. By engaging with PD-1 and CD80, PDL-1 contributes to suppression of the cancer-immunity cycle through inhibition of T-cell activation. CTLA-4 is expressed exclusively on T-cells and regulates the magnitude of T-cell activation during the initial phases of the immune response. Its primary function involves the inhibition of CD28, a key co-stimulatory receptor on T-cells [15]. The anti-PD-1/PDL-1 and anti-CTLA-4 mechanisms of action are described in Figure 1.

Unfortunately, not all patients respond favorably to these drugs. Clinical studies on the use of ICIs in BTC tumors as single agents have shown disappointing results. By contrast, the combination of ICIs with chemotherapy has emerged as the new standard of care in the first-line setting for patients with advanced or metastatic BTC.

### 3.1. ICIs in Advanced BTCs

#### 3.1.1. ICIs as Monotherapy or Doublet Therapy

Several studies have investigated the role of ICI therapy for advanced stages of BTC. The Phase Ib Keynote-028 trial included 24 patients with PDL-1-positive BTCs receiving pembrolizumab and reported an overall response rate (ORR) of 13% [12]. The Phase II Keynote-158 trial enrolled 104 patients with PDL-1-positive [defined as a combined positive score (CPS) ≥1%] (58.7%), PDL-1-negative (32.7%) and unknown (8.7%) BTCs receiving second- and subsequent-line therapy [13] with pembrolizumab, found an ORR of 5.8% and a median OS and PFS similar to previously published data (median OS and PFS of 6.2 and 4 months, respectively, with FOLFOX vs. active symptom control) [4]. In an attempt to evaluate whether PDL-1 could serve as an effective biomarker, these studies observed no significant differences in response rates according to PDL-1 status, Keynote-028 ORR 13% and Keynote-158 ORR 6.6% in the global population. A Phase II study of nivolumab monotherapy in the second-line setting also showed modest efficacy, with an ORR of 10.9% according to a central independent review. Notably, this particular study found that PDL-1 positivity was associated with an improved PFS [16]. However, PDL-1 status was not a sufficient biomarker for BTC.

The Phase II CA209-538 trial evaluated whether dual inhibition of PD-1 and CTLA-4 could achieve superior outcomes. This study enrolled 39 BTC patients who received nivolumab (3 mg/kg) plus ipilimumab (1 mg/kg) every 3 weeks for 4 cycles, followed by nivolumab every 2 weeks for up to 96 weeks or until progression or unacceptable toxicity [17]. Analysis of the BTC subgroup demonstrated an ORR of 23%, with a median PFS and OS of 2.9 and 5.7 months, respectively. Notably, responses were mainly observed in patients with iCCA and GB. All responders were microsatellite stable.

#### 3.1.2. ICIs in Combination with Chemotherapy

The combination of ICI and standard chemotherapy has shown the greatest success in the management of BTCs. A Phase II study investigated the benefits of combining cisplatin–gemcitabine with nivolumab for six cycles, followed by nivolumab maintenance in the absence of progression [11]. The study had a chemotherapy-resistant cohort and a metastatic disease, treatment-naïve cohort. The treatment-naïve cohort achieved a promising disease control rate (DCR) of 95%. The comparison between cohorts showed that chemotherapy-naïve patients had a longer median PFS than those with previous chemotherapy, although the difference was not significant. Analysis of 27 response-evaluable patients demonstrated that PDL-1 expression could not be used as a biomarker for predicting clinical response.

Preliminary results from a retrospective study found improved outcomes with the addition of anti-PD-1 antibodies to chemotherapy compared to anti-PD-1 therapy alone in the first-line setting [18]. A Phase II study enrolled 124 patients into three different arms (Arm 1, chemotherapy followed by chemotherapy plus durvalumab and tremelimumab; Arm 2, chemotherapy plus durvalumab; Arm 3, chemotherapy plus durvalumab and tremelimumab) with ORR as the primary endpoint [19]. Arms 2 and 3 presented higher response rates than Arm 1, while median PFS was similar across arms. The most common adverse events were nausea, pruritus, and neutropenia. Bacterial infections occurred in fewer than 20 of the 126 patients included. The most common grade 3 or 4 adverse events were hematological events. Chemotherapy plus durvalumab with or without tremelimumab had a manageable toxicity profile with fewer hematological and non-hematological side effects. Based on these findings, the combination of chemotherapy and durvalumab was selected for further evaluation in Phase III clinical trials. Table 1 shows the summary of safety data of the three main trials of ICI in combination with chemotherapy.

The TOPAZ-1 trial enrolled 685 patients with locally advanced unresectable or metastatic BTC who had not received prior systemic therapy for advanced disease. This trial randomized the participants to receive cisplatin–gemcitabine with durvalumab or placebo for eight cycles and subsequent maintenance with durvalumab/placebo, with OS as the primary endpoint. OS was significantly longer with durvalumab versus placebo, with median OS of 12.8 and 11.5 months, respectively. The estimated overall survival rates at 12-, 24-, and 36-month were 54.3%, 22.9% and 14.6% in the durvalumab group and 47.2%, 13.1% and 6.9% in the placebo group. Subgroup analysis revealed improved OS in patients with iCCA, good performance status, and Asian ethnicity. The durvalumab group also showed improvements in PFS and ORR [20]. In a recent follow-up analysis of BTC patients who received durvalumab plus gemcitabine–cisplatin were alive at 3 years compared with those receiving placebo plus gemcitabine–cisplatin. Median follow-up for the study was 41.3 months [21]. Notably, the addition of durvalumab did not significantly increase toxicity when compared to chemotherapy alone, with 99.4% and 98.8% of the durvalumab and placebo groups showing any grade adverse events, respectively. This similarity in adverse event rates confirms that durvalumab does not cause additional toxicity. The study also showed immune-mediated adverse event rates of 12.7% with durvalumab and 4.7% with placebo. Moreover, grade 3 or 4 immune-mediated adverse events occurred in 2.4% of the patients in the experimental arm.

Similar results were observed with first-line treatment pembrolizumab in combination with cisplatin and gemcitabine in the KEYNOTE-966 trial. This study showed a median OS and PFS comparable to those reported in the TOPAZ-1 trial (12.7 vs. 6.5 months, respectively). One of the main differences between the trials was that in KEYNOTE-966, patients continued maintenance pembrolizumab or placebo together with gemcitabine after eight cycles of complete treatment. At the final analysis, adverse events of any cause occurred in 524 of 529 (99%) participants in the pembrolizumab group and 532 of 534 (<100%) participants in the placebo group. The pembrolizumab arm showed manageable toxicity, with 369 (70%) developing grade 3 or 4 treatment-related adverse events, 102 (19%) discontinuing one or more of the study drugs, and 18 (3%) discontinuing all study drugs (Table 1). The survival benefit was similar for PDL-1-negative (CPS of <1%) and PDL-1-positive (CPS of ≥1%) cases [22].

Despite these advancements, identifying reliable biomarkers to predict treatment response remains an ongoing challenge in the field of immunotherapy in BTC. Current evidence suggests that PDL-1 expression alone may not sufficiently predict responses to ICI in this disease. Recently presented data on long-term survivors in the TOPAZ-1 trial revealed a higher prevalence of BRCA1/BRCA2 mutations among long-term survivors compared with non-long-term survivors in both treatment arms [23]. KRAS and Isocitrate dehydrogenase 1 (IDH1) mutations were more common in long-term survivors treated with durvalumab plus GemCis compared with the placebo group. However, prevalence rates of the mentioned alterations were low, and thus, this observation may require validation in a larger participant population. Further follow-up studies are needed to identify alternative biomarkers in order to better predict responses to the addition of ICIs to chemotherapy for BTCs.

The results for both TOPAZ-1 and KEYNOTE-966 have established ICIs, in combination with chemotherapy, as the new standard of care in first-line settings. Ongoing research is now focusing on developing ICIs for second- and further-line treatment strategies.

#### 3.1.3. ICI in Combination with Molecular Targeted Therapies

The development of molecularly targeted agents for BTCs has marked significant progress in recent years, displaying the need for comprehensive molecular testing for all unresectable or metastatic patients. Given the heterogeneity of biliary tract tumors, the anatomical location of each BTC subtype has specific clinical and molecular features. Fibroblast growth factor receptor 2 (FGFR2) and IDH1 mutations are almost exclusive to intrahepatic tumors, whereas HER2 alterations are more common in gallbladder tumors and eCCA [4]. Currently, molecular targeted therapies have been predominantly used in the second-line setting. Clinical trials have now begun to evaluate the benefit of combining ICI with small-molecule therapies.

- IDH1

IDH1 is predominantly expressed in hepatocytes, where it plays a critical role in lipid biosynthesis and maintenance of RedOx balance. Mutations in IDH1 result in abnormally high levels of the oncometabolite R-1-hydroxyglutarate, which acts as a competitive inhibitor of multiple enzymes implicated in epigenetics, DNA repair, metabolism, and other cellular processes [24]. Approximately 18–20% of patients with cholangiocarcinoma, primarily iCCA, present with IDH1 mutations [25]. Ivosidenib, a targeted therapy, has currently been approved for patients with IDH1 mutations with at least one prior line of systemic treatment [26]. To explore the potential benefits of adding ICIs in this population, the NCT04056910 trial is recruiting patients with IDH1-mutated tumors which progressed beyond standard treatment options who will receive nivolumab in combination with ivosidenib.

- FGFR2

FGFR2 serves as a receptor for the hormone fibroblast growth factor. It is a member of a gene family that comprises four receptor tyrosine kinases. Fusion or rearrangement of the FGFR2 gene is involved in tumorigenesis and tumor progression through constitutive activation of the receptor [24]. Around 13–14% of patients with BTC, mainly iCCA, present with FGFR2 fusion or other rearrangements [27]. Targeted therapies, in the form of oral tyrosine kinase inhibitors, have been approved for the treatment of BTC presenting FGFR2 fusions. These oral tyrosine kinase inhibitors have demonstrated an ORR ranging from 23% to 42%, depending on the specific agent [27]. However, acquired resistance to FGFR2 inhibitors has limited the effectiveness of these agents. Other next-generation inhibitors, such as the FGFR2 inhibitor RLY-4008 [28] and the multikinase inhibitor tinengotinib [29], are being evaluated for their potential to overcome acquired resistance to previous FGFR inhibitors. Additionally, early-phase trials are exploring immunotherapy combination approaches to enhance efficacy in this population. The FIGHT101, a three-part, open-label study, evaluated the safety, tolerability, and pharmacodynamics of pemigatinib, an FGFR2 inhibitor, in combination with pembrolizumab or retifanlimab in 23 patients with BTC. The study found that combination treatment showed no unexpected toxicities when compared to monotherapy [30].

- Mitogen-activated protein kinase (MAPK) kinase pathway

MEK inhibitors have traditionally been used in combination with BRAF inhibitors to enhance the blockade of the MAPK kinase growth signaling pathway in B-Raf-proto-oncogene (BRAF) V600E-mutant cancers [31]. Although the ROAR basket trial demonstrated promising results for this combination in BTC tumors [32], only 1–5% of patients with BTC present BRAF V600E mutation [33]. Immunotherapy has been investigated in conjunction with MEK inhibitors, such as cobimatinib. The rationale behind this combination is the observation that genomic alterations in the Ras/MAPK pathway correlate with lower levels of tumor-infiltrating lymphocytes. MAPK blockade may enhance T-cell function and antitumor activity, particularly when MEK inhibitors are combined with immunostimulatory drugs, like PD-1 or PDL-1 checkpoint inhibitors [34]. A Phase II study that evaluated atezolizumab alone or in combination with cobimatinib in BTC patients met its primary endpoint, showing an increase in PFS from 2.9 months with atezolizumab alone to 3.9 months with the combination treatment. The study provides evidence that MEK inhibitors may have some benefit in the context of systemic immunotherapy for BTCs, although the modest PFS benefit observed in the study reflects an unclear immunomodulation of the tumor microenvironment in this population [35]. The ongoing ETCTN-10476 Phase II randomized trial is evaluating the combination of atezolizumab plus varlilumab, a CD27 agonist, with or without cobimatinib. The addition of immune agonists may rescue T-cell function and could be an effective strategy to optimize the immunomodulatory potential of MEK inhibitors when used with ICI [36].

- Human epidermal growth factor receptor 2 HER-2

Her2 is a member of the type I receptor tyrosine kinase (RTK) family and functions as a proto-oncogene. By engaging with the extracellular region of type I RTKs, conformational changes occur that reveal the dimerization domain. This receptor dimerization activates the intracellular tyrosine kinase domain, leading to autophosphorylation and the initiation of a signaling cascade. Among RTKs, HER2 possesses the strongest catalytic activity. HER2 amplification/overexpression has been observed in approximately 4–8% of biliary tumors, principally in extrahepatic or gallbladder tumors [27], which is notably lower than the frequency observed in other gastrointestinal tumors. The KEYNOTE-811 trial evaluated the benefits of adding trastuzumab to pembrolizumab and chemotherapy in HER2-positive gastroesophageal junction or gastric tumors using intention-to-treat analysis, with the primary endpoints being PFS and OS [37]. Median OS was 20.0 and 16.9 months, and median PFS was 10.0 and 8.1 months in the pembrolizumab and placebo groups, respectively. Similar combinations are being evaluated in BTC (NCT05749900). Aside from monoclonal antibodies, other drugs targeting the HER2 family are being investigated. Furthermore, an ongoing study is currently evaluating the combination of tucatinib with trastuzumab, with or without oxaliplatin-based chemotherapy or pembrolizumab, in patients with unresectable or metastatic HER2-positive gastrointestinal cancer. In the initial safety and tolerability analysis, include patients with CCA and GB carcinoma (NCT04430738). We are waiting for the results in this cohort [38].

- Poly (ADP-ribose) polymerase inhibitors (PARPi)

Members of the PARP family are implicated in essential roles in cellular processes, including regulation of transcription, apoptosis, and the response to DNA damage. PARP1 is the most studied isoform. It possesses poly (ADP-ribose) activity and, when activated by DNA damage, catalyzes the formation of branched PAR chains to facilitate the recruitment of other repair proteins and facility the resolution of single-strand breaks [39]. PARP inhibitors have traditionally been used in the treatment of homologous repair-deficient cancers. Their application is based on the rationale of promoting DNA damage, which causes aberrant protein expression and may enhance immunogenicity. The Phase II BilT-02 study evaluated the effect of rucaparib in combination with immunotherapy as maintenance treatment following first-line chemotherapy in patients with advanced BTC tumors who showed no progression [40]. This trial did not meet the primary endpoint, with a PFS rate of 54.8% at 4 months, which was comparable to the 63% null estimate based on the ABC-02 trial [41]. While PARP inhibitors have emerged as a maintenance strategy after a platinum-based regimen in pancreatic cancer, as demonstrated in the POLO trial [42], the effectiveness of combining PARP inhibitors with immunotherapy in BTC requires investigation.

Interim results for the combination of pembrolizumab and olaparib in patients with advanced cholangiocarcinoma in the second-line setting showed a partial response (PR) in 1 patient and stable disease in 4 out of 12 patients, suggesting that this combination may be promising [43].

In summary, the integration of small-molecule inhibitors with ICI presents a promising strategy for enhancing the therapeutic response of BTCs harboring molecular alterations. However, the potential synergistic effects of these combinations need further investigation. Better understanding of the tumor microenvironment and molecular landscape of BTC, as well as the impact of the effect of targeted therapies and immunotherapies, may offer opportunities for improving outcomes in this challenging disease.3.1.4. ICI in Combination with Antiangiogenic Therapies

Angiogenesis, which refers to the formation of new blood vessels from pre-existing ones, is regulated by a delicate balance of proangiogenic and antiangiogenic factors [44]. Tumor-driven hypoxia upregulates proangiogenic factors, triggering the formation of new vessels that are crucial for tumor survival and proliferation. Vascular endothelial growth factor (VEGF) receptors are expressed not only on endothelial cells but also on tumor and immune cells. Proangiogenic factors play a key role in suppressing the antitumor immune response through various mechanisms, including impairment of antigen-presenting cells (APCs), enhancement of immunosuppressive cells such as regulatory T-cells and myeloid-derived suppressor cells, and inhibition of monocyte dedifferentiation and maturation into dendritic cells. Furthermore, those factors also inhibit progenitor cell differentiation into CD4+ and CD8+ lymphocytes, increasing T-cell exhaustion by upregulating the expression of PDL-1, CTLA-4, T-cell immunoglobulin and mucin domain-3 (TIM3), and Lymphocyte-activation gene 3 (LAG4) on T-cells. This VEGF-mediated immunosuppressive microenvironment may compromise the efficacy of ICI [45]. Specifically, VEGF overexpression is observed in around 40–70% of patients with advanced BTC and is associated with poor prognosis [46].

Several combinations of antiangiogenic agents and ICI therapies have been investigated. A Phase Ia/Ib study examined the combination of the anti-VEGFR2 antibody ramucirumab with pembrolizumab [47]. Although acceptable toxicity was observed, the efficacy of the combination did not differ much from that published in the FOLFOX trial in a second-line setting [48]. Lenvatinib, an antiangiogenic multikinase inhibitor, in combination with pembrolizumab has demonstrated promising antitumor activity with a manageable safety profile in patients with select advanced solid tumors. In fact, the LEAP-005 Phase II study demonstrated encouraging efficacy, with a median PFS and OS of 6.1 and 8.6 months, respectively [49]. The single-arm Phase II REGOMUNE trial evaluated the combination of avelumab plus regorafenib in BTC patients who had progressed to at least one prior line of systemic treatment [50]. The observed ORR of 13% did not meet the primary endpoint of 20% ORR. However, survival outcomes were quite promising, with a median PFS and OS of 2.5 and 11.9 months, respectively. The Phase II GEMINI-hepatobiliary trial (NCT05775159) is currently investigating volrustomig, a bispecific anti-PD-1/antiCTLA4, alone or in combination with lenvatinib or bevacizumab in hepatocellular carcinoma and BTC (Table 2).

**Figure 2:**
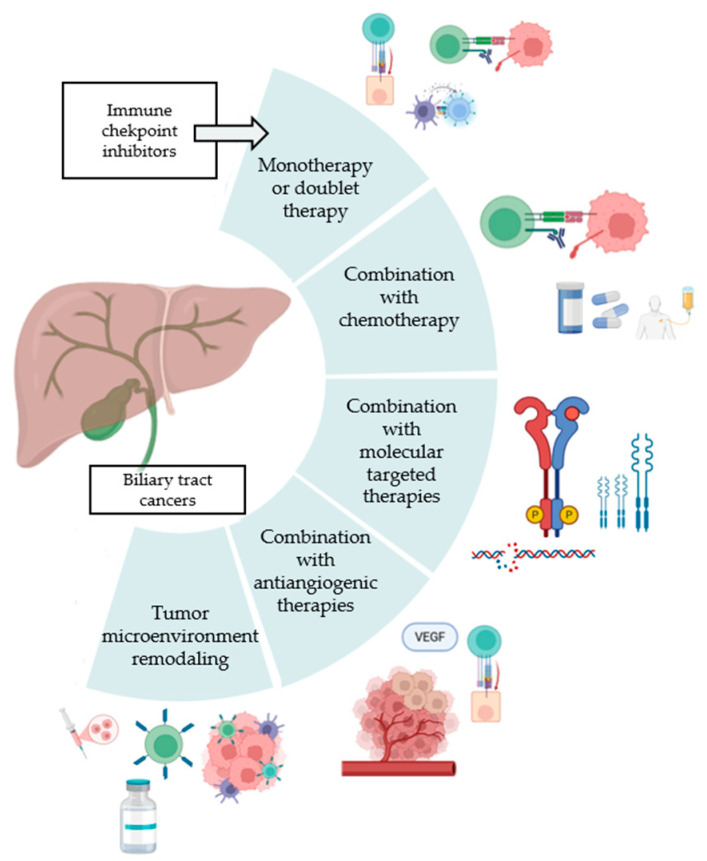
Targeting biliary tract cancer with immune-checkpoint inhibitor therapies and combinations.

Multiple studies have also evaluated the combination of antiangiogenics plus ICI and chemotherapy for BTC in first- and second-line settings. In the first-line setting, a Phase II study evaluating the combination of GEMOX with toripalimab and lenvatinib for advanced iCCA demonstrated promising outcomes, with an ORR of 80%, DCR of 94%, median PFS of 10 months, and median OS of 22.5 months. Grade ≥ 3 adverse events occurred in 57% of the patients, with neutropenia and leukocytopenia being the most common [51]. The IMBrave-151 Phase II trial randomized patients to GemCis–atezolizumab plus bevacizumab or placebo. The study found a median PFS of 8.4 and 7.9 months in the bevacizumab and placebo arms, respectively [52]. A biomarker analysis revealed that high VEGF gene expression was associated with improved PFS in patients who received bevacizumab [53]. The COMBATBIL trial evaluated the combination of FOLFOX plus atezolizumab plus bevacizumab among BTC patients in the second-line setting. The preliminary results of the study showed an ORR of 31.3%, with 28.5% of patients achieving a PR [54]. Moreover, the study found a median PFS and OS of 8.36 and 13.26 months, respectively. These results highlight the potential of combining antiangiogenic agents, ICIs, and chemotherapy to improve outcomes in patients with advanced BTC. Figure 2 shows the combinations of ICI in advanced BTC.

**Table 2:** Clinical outcomes of ICI in combination with antiangiogenic therapeutics.

Trial Name	Phase	Treatment Arm	Line of Therapy	Primary Endpoint	ORR (%)	PFS (Months)	OS (Months)	Reference
NCT02443324	Ia/Ib	Pembrolizumab + Ramucirumab	Second	Safety and tolerability	4	1.6	6.4	[47]
LEAP-005	II	Pembrolizumab + Lenvatinib	Second	Safety, ORR	10	6.1	8.6	[49]
REGOMUNE	II	Avelumab + Regorafenib	Second	ORR	13	2.5	11.9	[50]
NCT03951597	II	Toripalimab + Lenvatinib +GEMOX	First	ORR	80	10	22.5	[51]
IMBrave-151	II	Atezolizumab + GemCis + Bevacizumab/Placebo	First	PFS	24 B cohort 25 P cohort	8.4 B cohort 7.9 P cohort	14.9 B cohort 14.6 P cohort	[52]
COMBATBIL	Ib/II	Atezolizumab + Bevacizumab + FOLFOX	Second	ORR	31.3	8.36	13.26	[54]

ORR: Overall response rate; PFS: progression-free survival; OS: overall survival; GEMOX: gemcitabine–oxaliplatin; GemCis: Gemcitabine–Cisplatin; FOLFOX: Fluorouracil-Oxaliplatin; B: Bevacizumab; P: Placebo.

### 3.2. ICIs in Localized or Locally Advanced BTC

Only 20% of patients with BTCs are diagnosed with resectable disease [4]. However, the prognosis of these patients is still poor, with 5-year survival rates ranging from 10% to 30% [55]. In an effort to improve outcomes for a larger percentage of patients with resectable tumors, new treatment options are being evaluated in both adjuvant and neoadjuvant settings.

#### 3.2.1. Immunotherapy in the Adjuvant Setting

Postoperative recurrences are observed in 50% to 70% of resected BTCs [56]. Some studies report a predominance of distant metastases, and others report a higher rate of locoregional relapse [57,58]. Factors associated with an increased risk of relapse include R1 resection, lymph node metastases, and high serum levels of CA19-9 [59]. The influence of these factors may vary depending on tumor location. In iCCA, vascular invasion and elevated preoperative CA19.9 levels are independent risk factors for early recurrence. In contrast, perineural invasion and poor tumor differentiation are associated with early recurrence in eCCA.

The major Phase III studies on adjuvant treatment in BTC have shown disappointing results. The BCAT trial [57], which compared gemcitabine vs. surgery alone in patients with eCCA, and the PRODIGE12-ACCORD 18 Trial [60], which assessed the combination of gemcitabine–oxaliplatin vs. surgery alone, both showed negative results. The Japanese group also confirmed the benefit of S-1 compared with observation. The results demonstrated a 3-year overall survival rate of 67.7% in the observation group versus 77.1% in the S-1 group. This significant improvement in survival has established S-1 as a standard of care for resected BTC in Asian patients [61]. In current clinical practice, monotherapy with capecitabine for 6 months is the standard of care for resectable BTCs based on the findings of the Phase III BILCAP study [56]. The trial compared capecitabine (8 cycles started up to 12 weeks from surgery, with a maximum extension to 16 weeks from surgery) vs. surgery alone. Although the primary endpoint did not reach statistical significance, the intention-to-treat analysis of OS, adjusted for stratification factors and other prognostic factors such as nodal status, grade of disease, and sex, showed some benefits in the capecitabine arm. We are waiting for the results of the German ACTICCA-1 trial, which is evaluating the efficacy of gemcitabine and cisplatin vs. capecitabine in 440 patients with BTC after complete resection, with the primary endpoint of disease-free survival (NCT02170090). The search for more effective adjuvant therapies in BTC continues, with a focus on modern combinations incorporating ICIs or targeted therapy. The ARTEMIDE trial is investigating the combination of rilvegostomig, an anti-PD1/anti-TIGT bispecific monoclonal antibody, plus chemotherapy for patients with resected BTC [62]. The ADJUBIL trial is investigating the dual checkpoint inhibition approach (tremelimumab plus durvalumab) with or without capecitabine, with the primary endpoint of relapse-free survival (RFS) rate at 12 months [63]. The ACCORD trial (NCT04333927) is exploring the combination of camrelizumab plus chemoradiation with capecitabine vs. observation (no anticancer treatment until relapse) in high-risk patients with resectable tumors. Preliminary data from the BTC-CalenT study showed promising results with tislelizumab (anti-PD1 antibody) in combination with lenvatinib (a multikinase inhibitor) and capecitabine, with a 63% RFS rate at 1 year after completion of treatment in patients with resected BTC [64]. All these trials will help determine the utility of incorporating ICIs in this scenario.

#### 3.2.2. Immunotherapy in the Neoadjuvant Setting

The majority of patients are diagnosed with metastatic or locally advanced disease, characterized by local vascular invasion, involvement of both hepatic lobes, and nodal metastases. The risk of an incomplete resection (R1/R2) is significant, and these patients are usually considered inoperable. In this context, preoperative treatment is an appealing strategy. Several clinical trials have explored the effects of ICIs as a component of neoadjuvant therapy for BTCs [65].

The DEBATE trial investigated the efficacy of gemcitabine–cisplatin with or without durvalumab in patients with localized, potentially resectable BTC. After four cycles, patients were scheduled for surgery, followed by six cycles of durvalumab in both arms, with chemoradiotherapy being allowed in R1 or R2 tumors. The primary endpoint was R0 resection rate, and patients were randomized 2:1 to GemCis-durvalumab or GemCis alone. Sixty-eight percent of the patients in the experimental arm underwent surgical exploration, compared to 36% of those in the chemotherapy only arm, with R0 resection rates of 48% and 43%, respectively [66]. An ongoing study (NCT04506281) is evaluating the effects of an aggressive regimen combining toripalimab, lenvatinib, and chemotherapy as a neoadjuvant therapy for high-risk iCCA patients compared to no neoadjuvant treatment. All resected patients then receive adjuvant capecitabine as per the BILCAP regimen. The study population includes patients with tumor diameter >5 cm, vascular invasion by imaging, multiple tumor nodules, hilar lymph node metastases, or preoperative CA19-9 >200 U/mL. The primary endpoint is event-free survival. The DurGAP trial (NCT05640791) is investigating the effects of three cycles of neoadjuvant durvalumab plus chemotherapy (gemcitabine/nab-paclitaxel and cisplatin). Preliminary results have shown an ORR of 62.5%, with promising results in terms of PR having been demonstrated in 15 patients [67].

## 4. Future Directions

Although ICIs have shown good outcomes in the treatment of BTC, other immunotherapeutic approaches are also being explored. These novel strategies aim to harness the potential of the immune system through different mechanisms. Some studies in BTC have investigated the effects of bintrafusp α, a first-in-class bifunctional fusion protein formed by the extracellular domain of TFG-βRII and a human IG1 antibody. The extracellular domain of TFG-βRII has a dual mechanism, also working as a TGF-β trap, and thus allowing a potential inhibition of angiogenesis through TGF-β suppression, modulation of the stromal environment, and restoration of normal vascular homeostasis. Initial results from a Phase I trial involving 30 patients have demonstrated an ORR of 20% with a median PFS and OS of 2.5 and 12.7 months, respectively [68]. However, a more recent Phase II/III trial evaluating bintrafusp α in combination with chemotherapy has been discontinued prematurely based on the low likelihood of achieving its primary endpoint [69]. Agonist antibodies have been used to enhance the immune response. CD40 is a member of the tumor necrosis factor receptor family. CD40 is constitutively expressed on APCs, such as B cells and dendritic cells, as well as on T-cells and macrophages after cellular activation. CD40 agonist antibodies may enhance the immune response against tumors [67]. A currently recruiting study is investigating CDX1140, a CD40 agonist, in combination with capecitabine, oxaliplatin, and pembrolizumab in BTC patients [70]. Cancer vaccines represent another immunotherapeutic approach that is being explored for the treatment of BTC, with studies focusing on WT-1 and MUC1 antigens. Interestingly, 80% of BTCs presented WT-1 mutations. A Phase I trial exploring the combination of the vaccine plus gemcitabine in treatment-naïve patients with advanced BTC has demonstrated a 2-month DCR of 50% [71]. A Phase I trial of MUC1 peptide vaccination showed a good safety profile but limited efficacy, with only one out of eight patients achieving a response [72]. The effectiveness of cancer vaccines depends on two main factors: the function of the immune system and the selection of an appropriated target antigen. To enhance their efficacy, some strategies have been explored, including the incorporation of adjuvants, such as Toll-Like receptor agonists, and the use of advanced delivery systems like nanoparticles. These approaches may improve antigen presentation and immune activation [73]. Adoptive cell therapies have also been investigated, and a number of additional CAR-T-cell trials for BTC are currently ongoing [71]. Promising targets for CAR-T-cells include mesothelin, HER-2, and EGFR. Mesothelin, through its interaction with MUC16, contributes to metastatic progression and immune suppression; CAR-T-cells targeting mesothelin have demonstrated potential in reducing tumor burden [74]. Gallbladder tumors frequently express carcinoma embryonic antigen (CEA), and CEA-specific CAR-T-cells have demonstrated effective recognition and cytotoxic activity [75]. The clinical trial NCT03633773 is currently evaluating the safety and efficacy of MUC-1-directed CAR-T-cells in patients with iCCA in China. At present, clinical evidence remains insufficient to support the clinical use of CAR-T-cell therapy in advanced BTC. Further clinical trials are essential to establish its safety and effectiveness. We conducted a search of ongoing clinical trials in the first- and second-line settings, as well as in the adjuvant and neoadjuvant scenarios. We identified a total of 34 and 53 in first- and second-line settings in the metastatic context. Table 3 summarizes the clinical trials conducted in the adjuvant and neoadjuvant settings.

## 5. Conclusions

ICIs have now established their position in the first-line treatment of metastatic BTC, and novel combinations are under development. Available preclinical and clinical observations regarding the role of chemotherapy, targeted therapies, and antiangiogenic agents in modulating the immune microenvironment suggest that combining these approaches with ICI may result in a synergistic effect. Other therapies that harness the immune system beyond the more nuanced control of the immune response are currently in early development. The role of ICIs in the adjuvant and neoadjuvant treatment settings is also under investigation. Despite notably progress in recent years, BTC still bears a poor prognosis. To advance the field, ongoing research should prioritize next-generation sequencing testing to provide deeper insights into tumor genomics, uncover actionable mutations, and elucidate the mechanism of resistance that may guide more personalize therapeutic strategies. Additionally, the identification and validation of novel tumor-associated antigens is essential, as these could serve as targets for adoptive cell therapies, cancer vaccines, and other immunotherapeutic approaches—enhancing both specificity and efficacy. Addressing the heterogeneity of the TME, including stromal and immune cell components, may help overcome current therapeutic barriers and potentiate the effects of ICIs. Ongoing research into novel combinations and therapies is essential, and further advancements are needed to improve the strategies for patients with BTC.

## Figures and Tables

**Table 1:** Summary of safety data.

		NCT03046862		TOPAZ-1		KEYNOTE 966	
	ChT→ChT + D + T (n = 30)	ChT + D (n = 47)	ChT + S + T (n = 47)	D + cis + gem (n = 338)	Placebo + cis + gem (n = 342)	Pembro + ChT (n = 529)	Placebo + ChT (n = 534)
AE of any cause				336 (99.4%)	338 (98.8%)	524 (99%)	532 (<100%)
AE leading to withdrawal from any treatment	No	No	No	44 (13.0%)	52 (15.2%)	138 (26%)	122 (23%)
AE leading to death	No	No	No	12 (3.6%)	14 (4.1%)	31 (6%)	49 (9%)
Hematological							
Neutropenia	19 (59%)	12 (38%)	25 (53%)	107 (31.7%)	102 (29.8%)	321 (61%)	320 (60%)
Anemia	18 (56%)	13 (41%)	19 (40%)	163 (48.2)	153 (44.7%)	278 (53%)	269 (50%)
Thrombocytopenia	13 (41%)	3 (9%)	16 (34%)	43 (12.7%)	45 (13.2%)	199 (38%)	197 (36%)
Non-hematological							
Nausea	22 (69%)	32 (68%)	22 (48%)	136 (40.2%)	117 (34.2%)	233 (44%)	246 (46%)
Pruritus	22 (69%)	26 (55%)	25 (53%)	38 (11.2%)	28 (8.2%)	77 (15%)	51 (10%)
Anorexia	26 (81%)	21 (45%)	20 (43%)	-	-	144 (28%)	155 (29%)
Fatigue	21 (66%)	18 (38%)	20 (43%)	91 (26.9%)	90 (26.3%)	187 (36%)	172 (32%)
Fever	13 (41%)	15 (32%)	21 (44%)	68 (20.1%)	56 (16.4%)	-	-
Constipation	8 (25%)	23 (49%)	20 (43%)	108 (32%)	99 (28.9%)	186 (36%)	190 (36%)
AST/ALT elevation	2 (6%)	2 (6%)	4 (9%)	29 (8.6%) *	35 (10.2%) *	175 (33%)	209 (40%)
Asthenia	-	-	-	48 (14.2%)	48 (14.2%)	75 (15%)	95 (18%)
Immune-mediated AE							
Infusion reactions	-	-	-	-	-	117 (22%)	69 (13%)
Hypothyroid events	2 (6%)	4 (9%)	3 (6%)	20 (5.9%)	5 (1.5%)	46 (9%)	14 (3%)
Received systemic corticosteroids to manage immune AE	-	-	-	-	-	48 (9%)	26 (5%)
Pneumonitis	-	-	-	3 (0.9%)	2 (0.6%)	26 (5%)	10 (2%)
Colitis	-	-	-	2 (0.6%)	1 (0.3%)	9 (2%)	6 (1%)
Hepatitis	-	-	-	4 (1.2%)	2 (0.6%)	9 (2%)	7 (1%)
Pancreatic events	-	-	-	1 (0.3%)	2 (0.6%)	4 (1%)	6 (1%)
Dermatitis/rash	2 (5.3%)	0 (0%)	1 (5.3%)	12 (3.6%)	1 (0.3%)	10 (2%) **	3 (1%) **

ChT: chemotherapy; D: Durvalumab; T: Tremelimumab; Cis: cisplatin; Gem: gemcitabine; Pembro: Pembrolizumab; AE: adverse events; * Alanine aminotransferase increased ** Dermatitis.

**Table 3:** Ongoing neoadjuvant and adjuvant ICIs trials in BTC.

	Phase	Localization	Endpoint	Therapy	Enrolled *
**Neoadjuvant setting**					
NCT06721286	II	iCCA	1-year RFS	Teripalimab + cis/gem vs. surgery	70
NCT06341764	II	CCA	Recurrence rate of CCA	Cis/gem + Durvalumab + Tremelimumab	38
NCT04669496	II/III	iCCA	EFS	GEMOX + Lenvatinib + Toripalimab vs. surgery	178
**Adjuvant setting**					
NCT06997913	Observational	High-risk eCCA	2-year RFS	Tislelizumab + cape + Rt	10
NCT06717464	II	dCCA, hCCA, GB	1-year RFS	Toripalimab + cape	110
NCT05430698	II	pCCA LN+	12-month relapse-free survival rate	PD-1antibody plus GEMOX	62
NCT05239169	II/III	CCA	RFS at 12 months	D + T + cape	40
NCT04782804	I/II	iCCA high risk	RFS	Tisle + cape vs. cape	30
NCT04333927	II	eCCA high risk	OS	Camrelizumab + cape + Rt vs. observation	92
NCT03820310	II	iCCA	PFS and 2-year survival	Traditional therapy plus autologous TCM cellular immunotherapy vs. traditional therapy	20

iCCA: intrahepatic cholangiocarcinoma; dCCA: distal cholangiocarcinoma; pCCA: perihilar cholangiocarcinoma; LN+: lymph nodes positive; Cis/Gem: cisplatin/gemcitabine; Cape: capecitabine; D: durvalumab; T: tremelimumab; Rt: Radiotherapy; Tisle: Tislelizumab. OS: overall survival; RFS: recurrence-free survival; EFS: event-free survival. * estimated.

## Abbreviations

The following abbreviations are used in this manuscript:

BTC	biliary tract cancer
ICI	immune-checkpoint inhibitors
iCCA	intrahepatic cholangiocarcinoma
eCCA	extrahepatic cholangiocarcinoma
GB	gallbladder cancer
CCA	cholangiocarcinoma
FDG-PET	^18^F-2-fluoro-2-deoxy-d-glucose-positron emission tomography
OS	overall survival
PD-1/PDL-1	programmed cell death protein-1/ligand-1
MSI	microsatellite instability
TMB	tumor mutational burden
TME	tumor microenvironment
FDA	food and drug administration
PFS	progression-free survival
TP53	tumor protein p53
BRCA2	breast cancer gene
Chemo-IO	chemo-immunotherapy
KRAS	Kirsten rat sarcoma
ARID1	at-rich interaction domain 1A
CTLA-4	cytotoxic T-lymphocyte antigen-4
ctDNA	circulating tumor DNA
ORR	overall response rate
CPS	combined positive score
DCR	disease control rate
IDH1	isocitrate dehydrogenase 1
FGFR2	fibroblast growth factor receptor 2
MAPK	mitogen-activated protein kinase
MEK	methyl ethyl ketone
BRAF	B-Raf-proto-oncogene
Her2	Human epidermal growth factor receptor 2
RTK	receptor tyrosine kinase
PARPi	Poly (ADP-ribose) polymerase inhibitors
PR	partial response
VEFG	vascular endothelial growth factor
APC	antigen-presenting cells
TIM3	T-cell immunoglobulin and mucin domain-3
LAG4	lymphocyte-activation gene 3
RFS	relapse-free survival
CEA	carcinoma embryonic antigen
